# Supplementary material for: The Chlamydia pneumoniae effector SemD exploits its host’s endocytic machinery by structural and functional mimicry
Source: Nat Commun. 2024 Aug 24;15:7294. doi: 10.1038/s41467-024-51681-3 (PMC11344771; doi:10.1038/s41467-024-51681-3)

Supplementary Information

The C*hlamydia pneumoniae* effector SemD exploits its host's endocytic machinery by structural and functional mimicry

Kocher et al.

Supplementary Content

Supplementary Figures.. 2

Supplementary Tables.. 13

Supplementary References 19

Uncropped Scans of gels and blots 20

Supplementary Figures

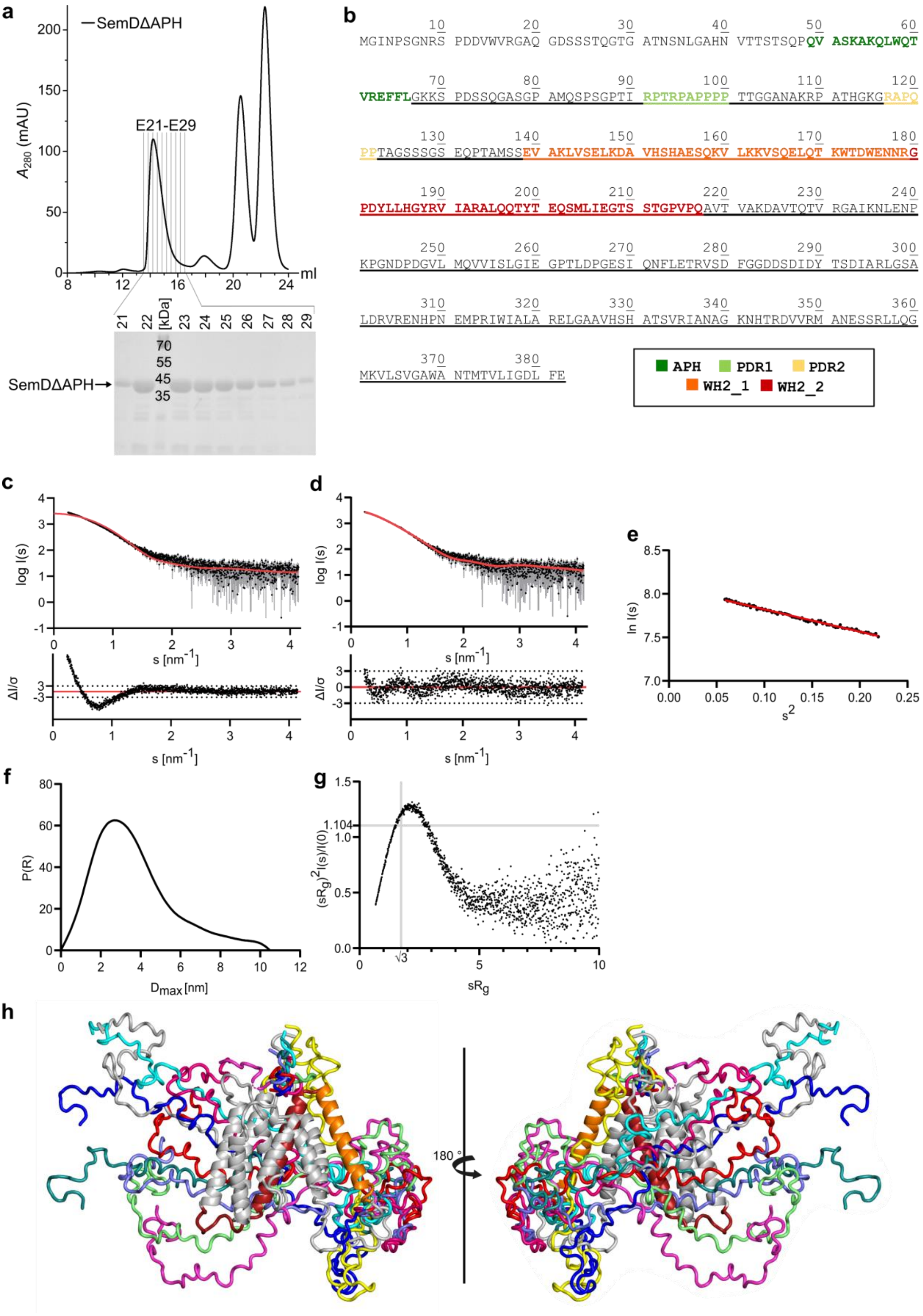

Supplementary Figure 1 │ SemDΔAPH purification and SAXS analysis. a SEC of SemDΔAPH (*top*) in PBS, separated on a HiLoad 16/600 Superdex 200 pg column. From elution fractions 21-29, SDS samples were prepared and loaded on an SDS gel, stained with Coomassie brilliant blue (*bottom*). **b** Amino acid sequence of full length SemD (residues 1-382). The underlined fragment corresponds to SemDΔAPH (residues 67-382). Individual domains are marked: amphipathic helix (APH, darkgreen), proline-rich domains (PRD1: bright green, PRD2: yellow), WH2_1 (orange) and WH2_2 (red). **c** Theoretical scattering data of the SemDΔAPH crystal structure. Experimental data are shown in black dots, with grey error bars. The theoretical scattering fit (χ^2 value of 14.63) created with CRYSOL, is shown as red line and below is the residual plot of the data. **d** Scattering data of SemDΔAPH. Experimental data are shown in black dots, with grey error bars. The best CORAL model fit (χ^2 value of 1.197) is shown as red line and below is the residual plot of the data. **e** The Guinier plot of SemDΔAPH showed a stable Guinier region with a R_g of 2.78 nm. **f** The *p(r)* function of SemDΔAPH showed a globular molecule with an elongated part and a D_{max} value of 10.49 nm. **g** Dimensionless Kratky plot of SemDΔAPH showed an elongated particle. Source data for **c-g** are provided as Source Data file. **h** Overlay of ten independent CORAL models with a χ^2 value ranging from 1.197 – 2.098. The SemDΔAPH crystal structure shown in cartoon representation was used as template and the missing residues were modelled with CORAL and shown as loops. The grey model (χ^2 value of 1.197) is shown in detail in Fig. 1c.

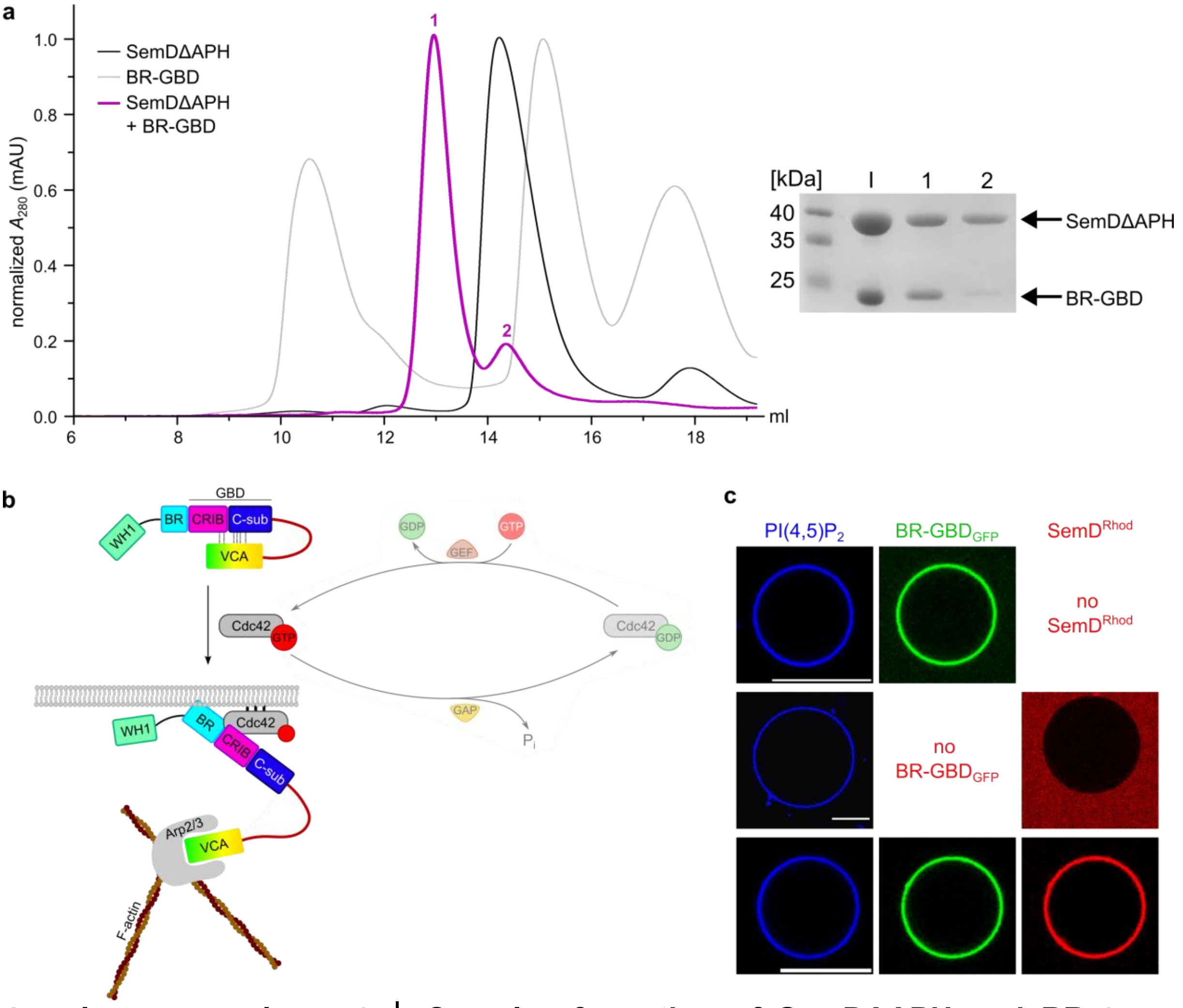

Supplementary Figure 2 │ Complex formation of SemDΔAPH and BR-GBD. **a** SEC (*left*) for SemDΔAPH (black), BR-GBD (grey) and SemDΔAPH + BR-GBD (magenta). Protein composition of peak 1 and 2 were analysed on an SDS gel (*right*), stained with Coomassie brilliant blue. The gel was loaded from left to right with the SEC-input sample (I), Peak 1 (1) and Peak 2 (2). **b** Schematic representation of N-WASP activation by $Cdc42_{GTP}$. **c** Confocal images of PI(4,5)P_2-GUVs with rhodamine-labelled SemD ($SemD^{Rhod}$) and $BR\text{-}GBD_{GFP}$. (Scale bars 10 µm).

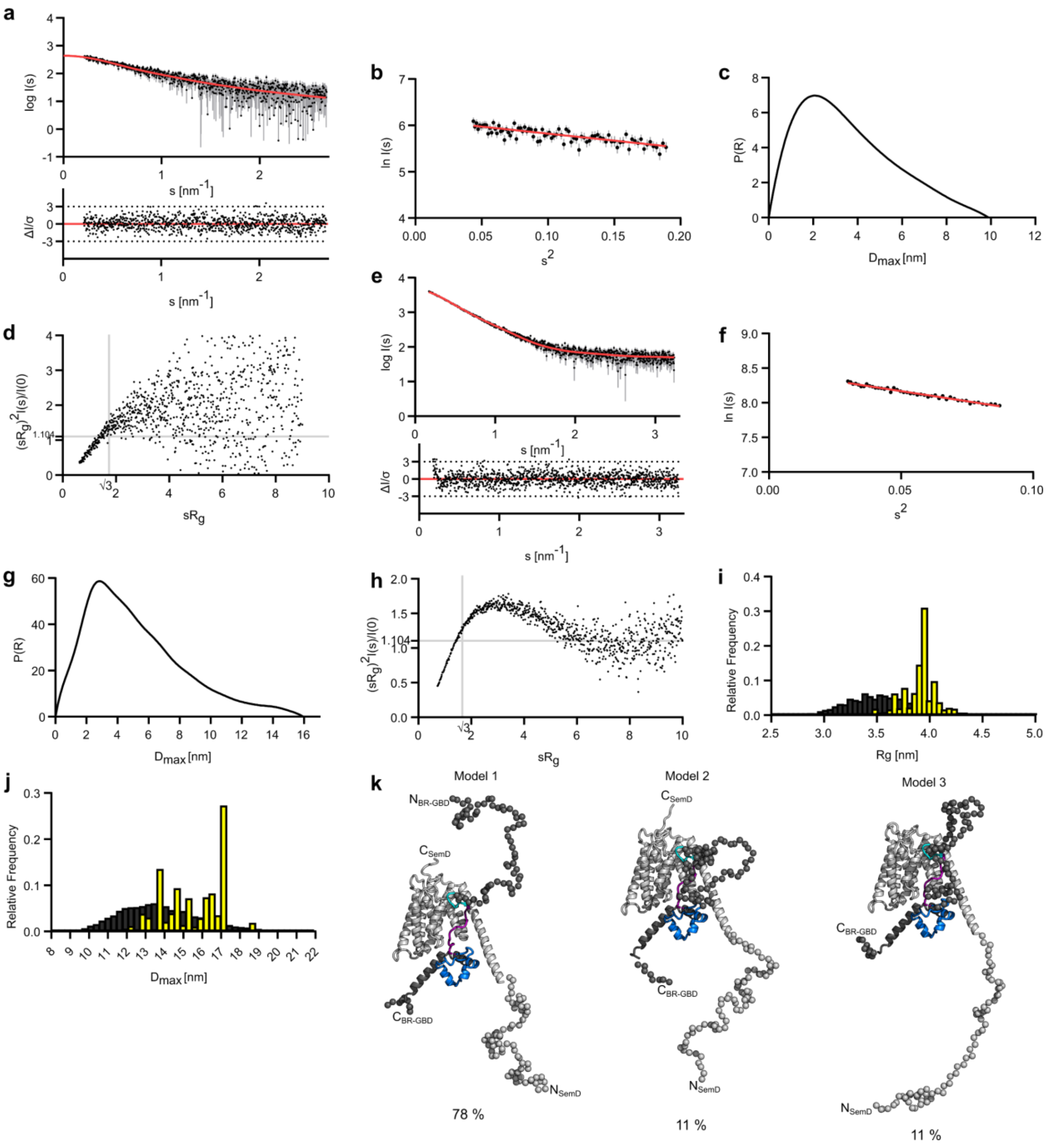

Supplementary Figure 3 │ Small-angle X-ray scattering data from BR-GBD alone and SemDΔAPH in complex with BR-GBD. a Scattering data of BR-GBD. Experimental data are shown in black dots, with grey error bars. The GNOM fit is shown as red line and below is the residual plot of the data. **b** The Guinier plot of BR-GBD showed a stable Guinier region with a R_g of 3.01 nm. **c** The *p(r)* function of BR-GBD showed a globular molecule with an elongated part and a D_{max} value of 9.90 nm.

d The Dimensionless Kratky plot showed a high degree of flexibility for BR-GBD apo. **e** Scattering data of SemDΔAPH in complex with BR-GBD. Experimental data are shown in black dots, with grey error bars. The EOM ensemble model fit is shown as red line and below is the residual plot of the data. **f** The Guinier plot of SemDΔAPH in complex with BR-GBD showed a stable Guinier region with a R_g of 4.19 nm. **g** The *p(r)* function of SemDΔAPH in complex with BR-GBD showed an elongated particle with a D_{max} value of 15.94 nm. **h** The Dimensionless Kratky plot of SemDΔAPH in complex with BR-GBD showed an elongated particle with a high degree of flexibility. **i&j** R_g and D_{max} distribution of SemDΔAPH in complex with BR-GBD. Ensemble pool is shown in grey, selected EOM models are shown in yellow. The EOM analysis showed that the assembled complex (in blue) favoured a more extended conformation in comparison to the random pool (in grey). Source data for **a-j** are provided as Source Data File. **k** Selected EOM models from SemDΔAPH in complex with N-WASP. The used parts from the crystal structure of SemDΔAPH in complex with N-WASP are shown in cartoon representation and the missing termini parts in spheres. On the left site Model 1 with a volume fraction of 78 % is shown, in the middle and on the right are Model 2 and 3 with a volume fraction of 11 % each.

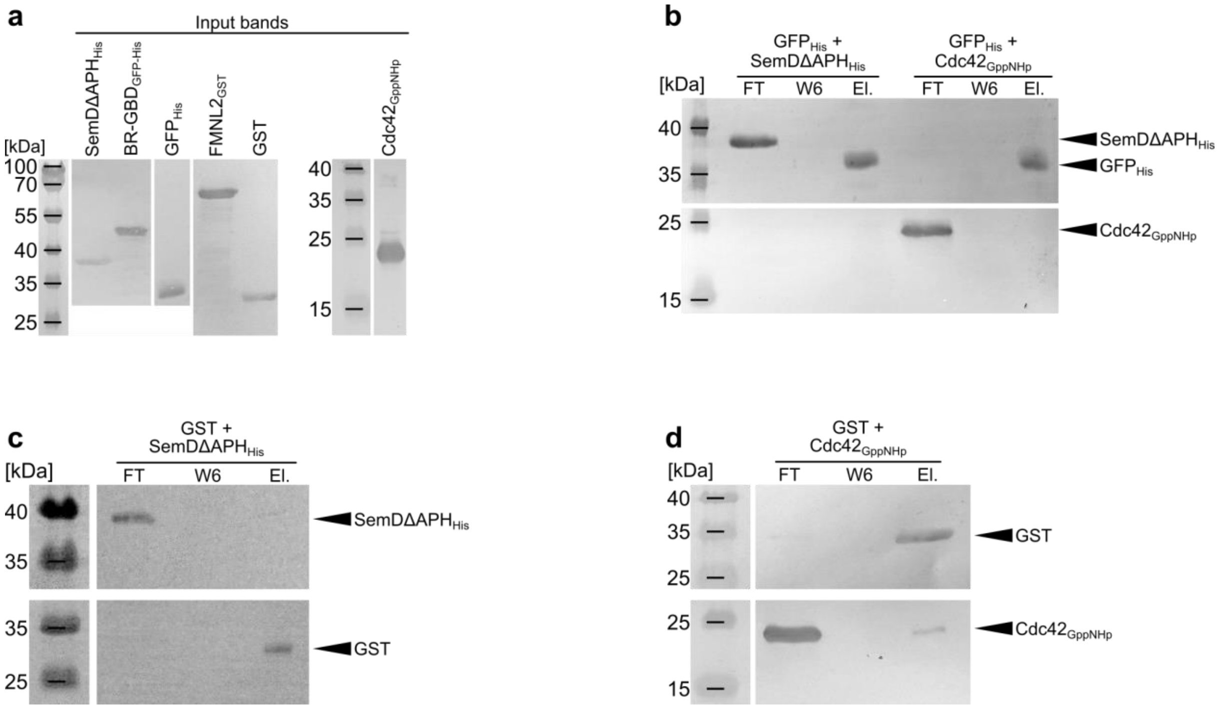

Supplementary Figure 4 │ Negative controls for pulldown assays from Figure 4. **a** Input bands for the recombinant and tagged proteins used for pulldown experiments from Fig. 4. **b** Negative control for GFP-Trap® pulldown experiments from Fig. 4a. SemDΔAPH$_{His}$ and Cdc42$_{GppNHp}$ were tested against GFP$_{His}$. flow-through (FT), W6 and Elution (El) were fractionated by SDS/PAGE and probed with anti-His for GFP$_{His}$ and SemDΔAPH$_{His}$ and anti-Cdc42 antibodies for Cdc42$_{GppNHp}$. **c,d** Negative control for GST-pulldown from Fig. 4e. SemDΔAPH$_{His}$ and Cdc42$_{GppNHp}$ were tested against GST. flow-through (FT), W6 and Elution (El) were fractionated by SDS/PAGE and probed with anti-GST for GST, anti-His for SemDΔAPH$_{His}$ and anti-Cdc42 antibodies for Cdc42$_{GppNHp}$. Uncropped blots are provided as Source Data file.

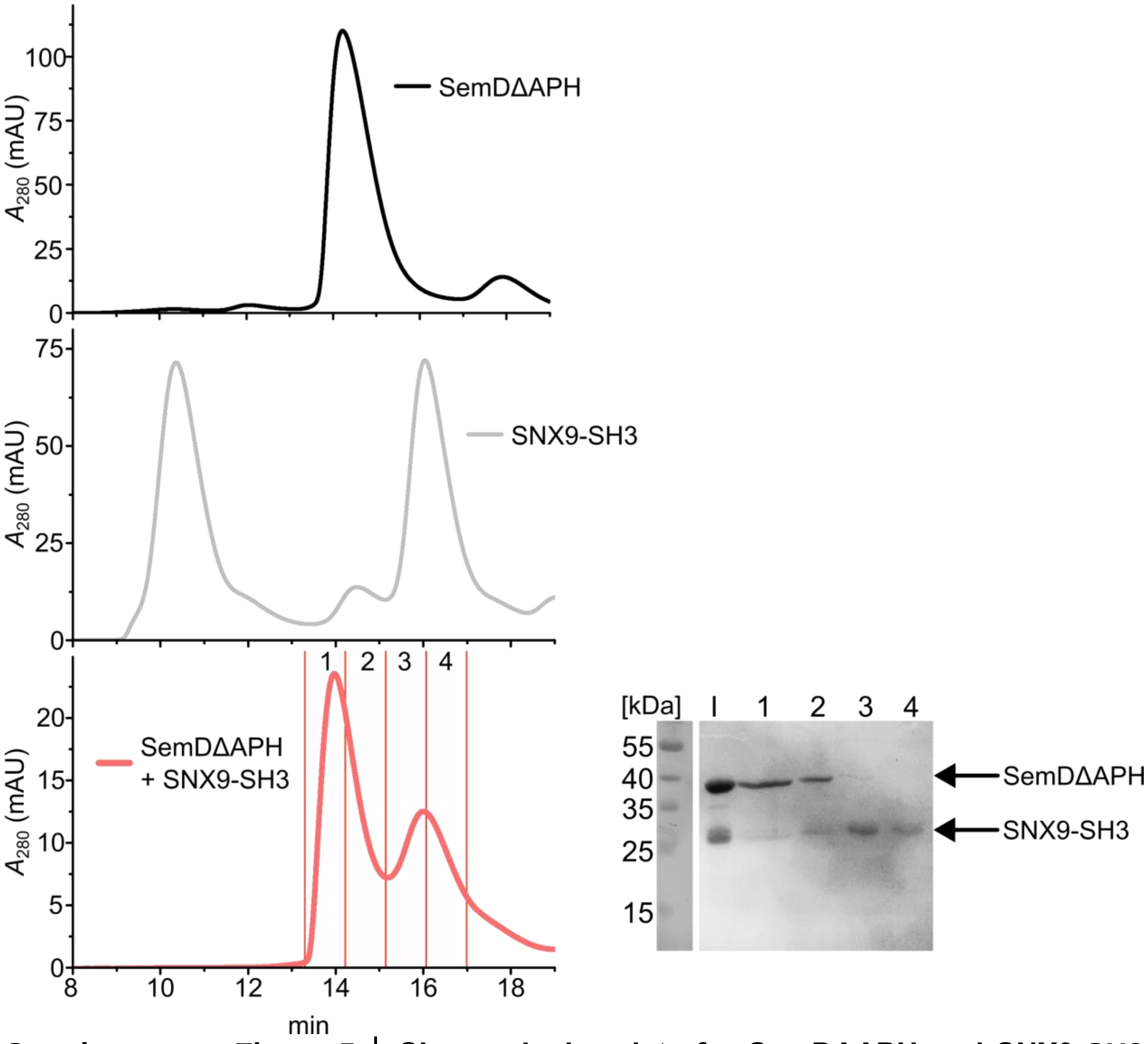

Supplementary Figure 5 │ Size exclusion data for SemDΔAPH and SNX9-SH3 complex purification. SEC for SemDΔAPH (top, black), SNX9-SH3 (middle, grey) and SemDΔAPH + SNX9-SH3 (bottom, red). For the elution fractions 1 to 4, SDS samples were prepared and the protein composition was analysed on an SDS gel (right), stained with Coomassie brilliant blue. The gel was loaded from left to right with the SEC-input sample (I) and elutions 1-4.

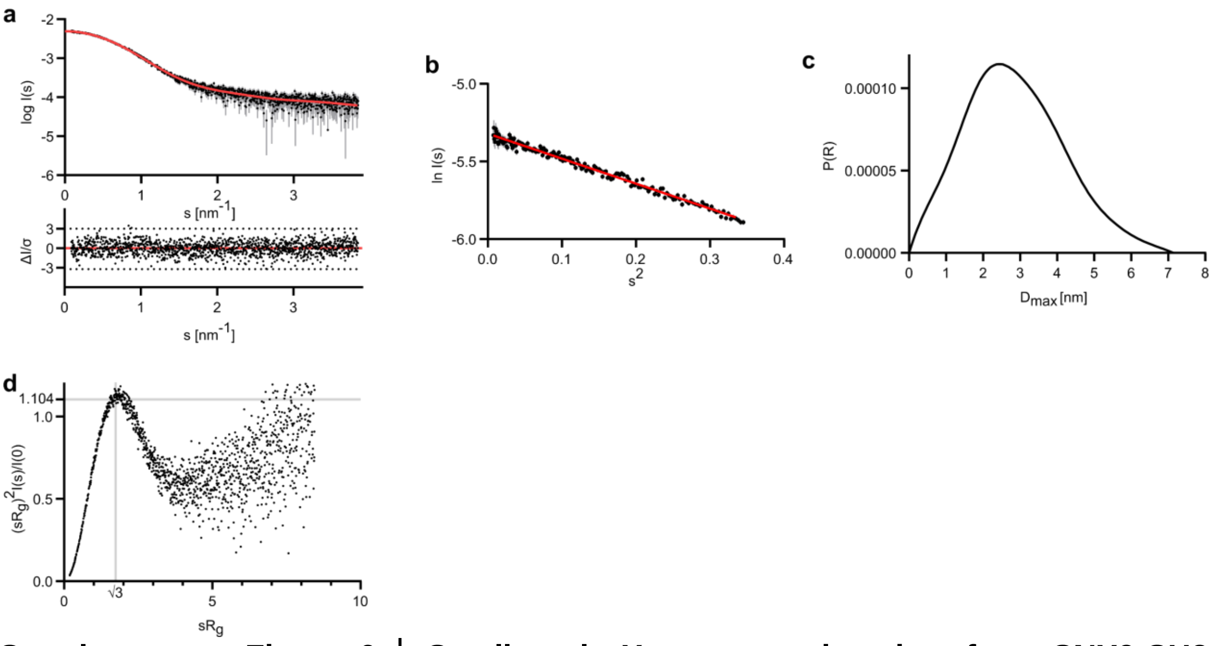

Supplementary Figure 6 │ Small-angle X-ray scattering data from SNX9-SH3 alone. a Scattering data of SNX9-SH3 alone. Experimental data are shown in black dots, with grey error bars. The GNOM fit is shown as red line and below is the residual plot of the data. **b** The Guinier plot of SNX9-SH3 showed a stable Guinier region with a R_g of 2.20 nm. **c** The *p(r)* function of SNX9-SH3 apo showed a globular molecule with an elongated part and a D_{max} value of 7.14 nm. **d** The Dimensionless Kratky plot of SNX9-SH3 showed a compact globular molecule containing flexible parts. Source data for **a-d** are provided as Source Data file.

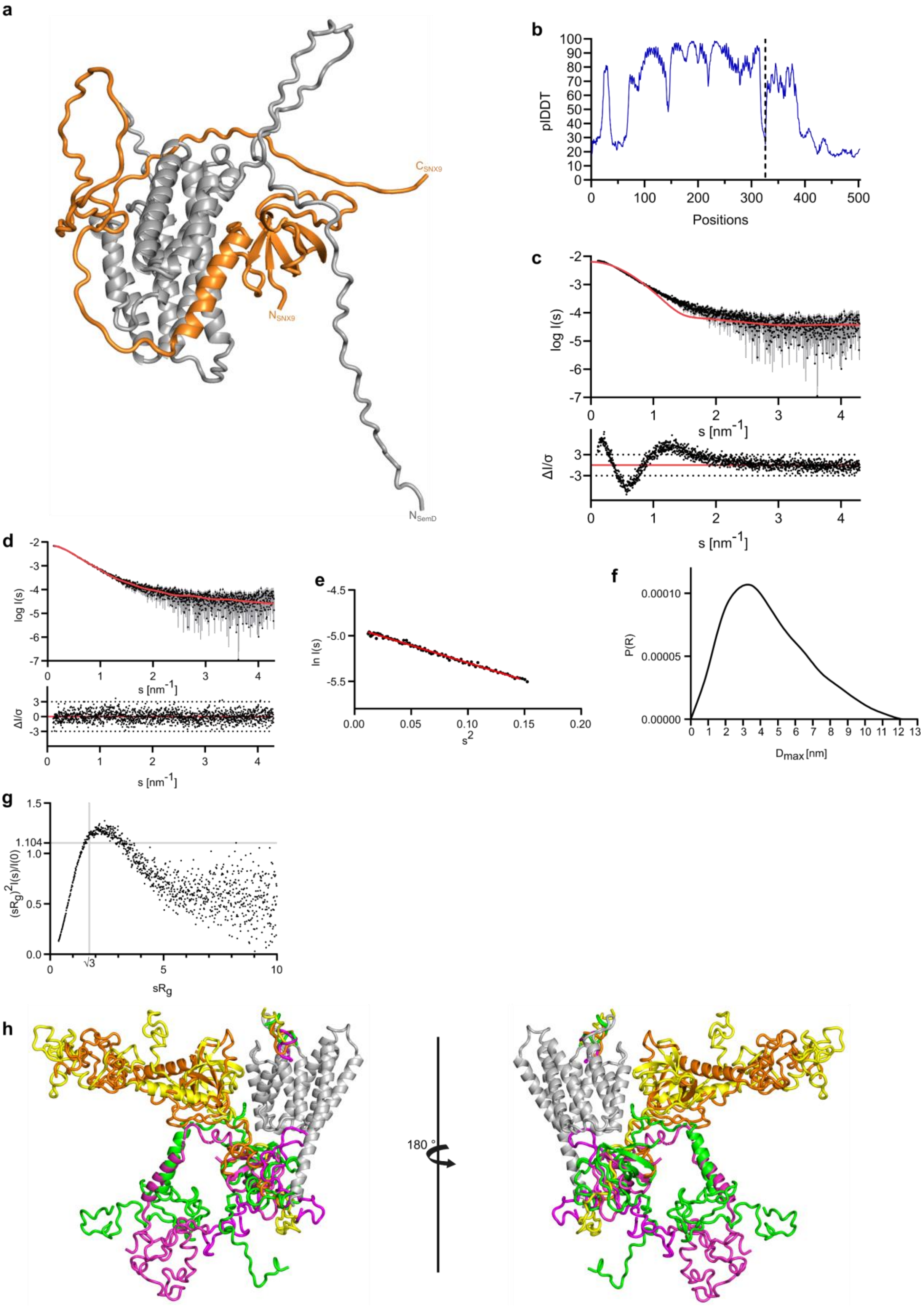
a
C$_{SNX9}$
N$_{SNX9}$
N$_{SemD}$
b
pIDDT
Positions
c
log I(s)
s [nm^{-1}]
ΔI/σ
s [nm^{-1}]
d
log I(s)
s [nm^{-1}]
ΔI/σ
s [nm^{-1}]
e
ln I(s)
s^2
f
P(R)
D$_{max}$ [nm]
g
(sR$_g$)^{2}I(s)/I(0)
1.104
√3
sR$_g$
h
180 °

Supplementary Figure 7 │ Small-angle X-ray scattering data from SemDΔAPH in complex with SNX9-SH3. a An AlphaFold2 prediction of SemDΔAPH (grey) in complex with SNX9-SH3 (orange). **b** pLDDT score from the AlphaFold2 prediction. The left part corresponds to SemDΔAPH and the right part to SNX9-SH3. **c** Theoretical scattering of the SemDΔAPH in complex with SNX9-SH3 from AlpfaFold2 prediction. Experimental data are shown in black dots, with grey error bars. The theoretical scattering fit (χ^2 value of 8.49) created with CRYSOL, is shown as red line and below is the residual plot of the data. **d** The experimental Scattering data of SemDΔAPH in complex with SNX9-SH3 are shown in black dots, with grey error bars. The best CORAL model fit (χ2 value of 1.018) is shown as red line and below is the residual plot of the data. **e** The Guinier plot of SemDΔAPH in complex with SNX9-SH3 showed a stable Guinier region with a *Rg* of 3.39 nm. **f** The *p(r)* function of SemDΔAPH in complex with SNX9-SH3 showed an elongated molecule and a D_{max} value of 12.16 nm. **g** The Dimensionless Kratky plot of SemDΔAPH in complex with SNX9-SH3 apo showed a compact globular molecule still containing flexible elements. Source data for **a-g** are provided as Source Data file. **h** Overlay of 4 independent CORAL models with a χ^2 value ranging from 1.018 – 1.092. The SemDΔAPH crystal structure (shown in grey) and the AlphaFold2 docking interface from SNX9-SH3 with the N-terminal part of SemDΔAPH, shown in cartoon representation were used as templates and the missing residues as well as the orientation were modelled with CORAL. The orange model (χ2 value of 1.018) is shown in detail in Fig. 5d.

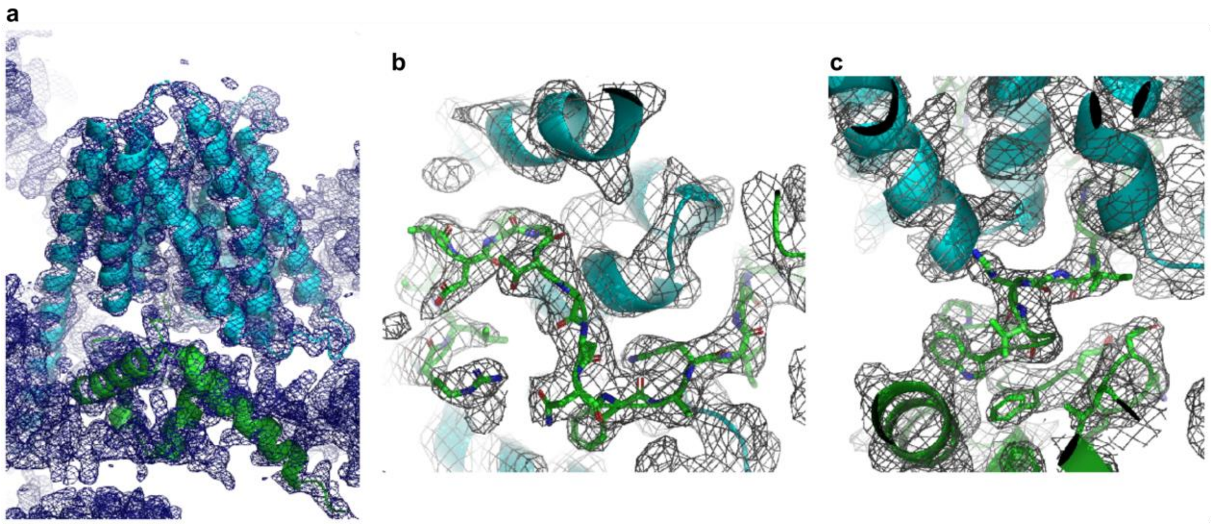

Supplementary Figure 8 │ Electron density of the SemD – BR-GBD interface. a Shown is the electron density of the overall structure of SemD (highlighted as blue cartoon) and BR-GBD (highlighted as green cartoon). **b,c** Zoom in on the interaction interface. For clarity reasons the SemD structure is shown as cartoon, whereas the BR-GBD protein is shown as sticks. The electron density (2F0-Fc) is shown as grey mesh, contoured at 1.0 sigma. Although the structure is of medium resolution, the side chains of BR-GBD are clearly visible in the electron density.

Supplementary Tables

Supplementary Table 1. Data collection and refinement statistics of the crystal structure of SemDΔAPH and SemDΔAPH - BR-GBD complex

	**SemDΔAPH**	**SemDΔAPH + BR-GBD**
**Data collection**		
Space group	P1	P 31 2 1
Cell dimensions		
a, *b*, *c* (Å)	46.99 51.95 58.62	120.22 120.22 65.24
α, β, γ (°)	88.33 105.89 117.43	90.0 90.0 120.0
Resolution (Å)	45.82 - 2.1 (2.175 - 2.1)*	52.06 - 3.3 (3.418 - 3.3) *
R_{sym} or R_{merge}	0.07588 (0.5214)	0.1328 (0.8603)
I / σI	10.61 (2.56)	17.63 (3.40)
Completeness (%)	97.65 (97.52)	99.66 (99.65)
Redundancy	3.5 (3.6)	20.7 (21.8)
**Refinement**		
Resolution (Å)	45.82 - 2.1 (2.175 - 2.1)*	52.06 - 3.3 (3.418 - 3.3) *
No. reflections	26816 (2675)	8408 (852)
R_{work} / R*free*	0.2195 /0.2745	0.2277 /0.2439
No. atoms		
Protein	3560	2617
Ligand/ion	0	0
Water	216	0
B-factors	(Ask for input)	
Protein	38.76	92.85
Ligand/ion	-	-
Water	40.8	-
R.m.s. deviations		
Bond lengths (Å)	0.011	0.012
Bond angles (°)	1.19	1.68

*Data collection resulted in both dataset from a single crystal. *Values in parentheses are for highest-resolution shell.

Supplementary Table 2. Overall SAXS Data of SemDΔAPH, BR-GBD, SNX9-SH3 and the corresponding complexes

**Data collection parameters**					
SAXS Device	P12, PETRA III, DESY Hamburg[1]				
Detector	PILATUS 6 M (423.6 x 434.6 mm^2)				
Detector distance (m)	3.0				
Beam size	120 µm x 200 µm				
Wavelength (nm)	0.124				
Sample environment	Quartz glass capillary, 1 mm ø				
Absolute scaling method	Comparison with scattering from pure H_2O				
Normalization	To transmitted intensity by beam-stop counter				
Scattering intensity scale	Absolute scale or relative scale, cm^{-1}				
s range (nm^{-1})‡	0.03 – 7.0				
**Sample**	**SemDΔAPH apo**	**BR-GBD apo**	**SemDΔAPH + BR-GBD**	**SNX9-SH3 apo**	**SemDΔAPH + SNX9 SH3**
Organism	*Chlamydia pneumoniae GiD*[2]				
UniProt ID (range)	Q9Z7M7 (67-382)	O08816 (142-273)		Q9Y5X1 (1-160)	
Mode of measurement	Online SEC-SAXS				
SEC-Column	Superdex200 increase 10/300 GL				
Flowrate (ml/min)	0.6				
Injection volume (µl)	100				
Temperature (°C)	20				
Exposure time (# frames)	0.995 s (2400)				
# frames used for averaging	19	22	17	46	63
Protein buffer	PBS (137 mM NaCl, 2.7 mM KCl, 10 mM Na_2HPO_4, 1.8 mM KH_2PO_4), pH 8.5, 3% Glycerol				
Protein concentration (mg/ml)	8	10	8	3.3	3.3

**Structural parameters**					
Guinier Analysis (PRIMUS)					
I(0) ± σ (cm^{-1})	3224.17 ± 9.92	456.12 ± 13.03	4722.19 ± 35.06	0.0049 ± 0.000014	0.0073 ± 0.00002
R_g ± σ (nm)	2.78 ± 0.01	3.01 ± 0.13	4.19 ± 0.04	2.20 ± 0.01	3.39 ± 0.02
s-range (nm^{-1})	0.243 – 0.468	0.209 – 0.432	0.173 – 0.295	0.087 – 0.579	0.110 – 0.382
min < sRg < max limit	0.67 – 1.30	0.63 – 1.30	0.74 – 1.24	0.19 – 1.27	0.37 – 1.29
Data point range	1 - 82	1 - 81	1 - 45	1 - 170	1 - 95
Linear fit assessment (R^2)	0.99	0.64	0.98	0.98	0.99
PDDF/P(r) Analysis (GNOM)					
I(0) ± σ (cm^{-1})	3279.00 ± 9.82	434.70 ± 8.56	4667.00 ± 34.88	0.0049 ± 0.000012	0.0074 ± 0.00003
R_g ± σ (nm)	2.95 ± 0.01	2.91 ± 0.06	4.29 ± 0.05	2.23 ± 0.007	3.53 ± 0.02
D_{max} (nm)	10.49	9.90	15.94	7.14	12.16
Porod volume (nm^3)	67.39	19.49	102.29	41.60	93.81
s-range (nm^{-1})	0.243 – 4.142	0.212 – 2.682	0.173 – 3.236	0.087 – 3.845	0.110 – 4.30
χ2 / CorMap P-value	0.925 / 0.081	1.013 / 0.579	1.070 / 0.234	0.972 / 0.076	0.977 / 0.759
**Molecular mass (kDa)**					
From *I*(0)	n.d.	n.d.	n.d.	n.d.	n.d.
From Qp[3]	38.03	15.69	60.09	16.44	52.51
From MoW2[4]	37.53	18.91	48.24	16.79	46.93
From Vc[5]	37.22	16.79	43.03	21.95	49.44
Bayesian Inference[6]	36.90	18.05	45.68	18.68	49.78
From sequence	35.13	19.74	54.87	18.80	53.90

**Rigid body modeling**					
CORAL					
Symmetry	P1				P1
s-range for fit (nm^{-1})	0.243 – 4.142				0.110 – 4.30
χ^2, CorMap *P*-value	1.197 / 0.005				1.018 / 0.507
EOM					
Symmetry			P1		
s-range for fit (nm^{-1})			0.173 – 3.236		
χ^2, CorMap *P*-value			1.107 / 0.033		
**SASBDB accession codes[7]**	SASDTQ5	SASDTR5	SASDTU5	SASDTS5	SASDTT5
**Software**					
ATSAS Software Version[8]			3.0.5		
Primary data reduction			CHROMIXS[9] / PRIMUS[10]		
Data processing			GNOM[11]		
Rigid body modelling			CORAL[12]		
Flexibility ensemble modelling			EOM[13,14]		
Structure validation			CRYSOL[15]		
Statistic goodness-of-fit test			χ^2, CorMap[16]		
Model visualization			PyMOL[17]		

‡s = 4πsin(θ)/λ, 2θ – scattering angle, n.d. not determined

Supplementary Table 3. PCR Primers

Internal number	Name	Sequence (5' -3')
**C-4130**	Flag-BR_CRIB-TEV Hin	TAGAAATAATTTTGTTTAACTTTAAGAAGGAGATATACATATGGATTACAAAGACGATGACGATAAGGATTACAAAGACGATGACGATAAGTCTGAAAAAAGACGAGATGCTC
**C-4131**	Flag-BR_CRIB-TEV Her	CTCAATGGTGATGGTGATGATGGTGGTGATGGTGCTCGAGTCCTTGAAAATACAAGTTTTCTGCTTGCCTTCGGAGTTCAT
**C-4214**	CPn0677_co 67-382 in pSL4 hin	AAATAATTTTGTTTAACTTTAAGAAGGAGATATACATATGGGTAAGAAAAGCCCGGATAG
**C-4215**	CPn0677_co 67-382 in pSL4 her	CCGGATCTCAATGGTGATGGTGATGATGGTGGTGATGGTGTTCGAACAGATCACCAATCAG
**C-4364**	SNX9_SH3 in pSL4 hin	AAATAATTTTGTTTAACTTTAAGAAGGAGATATACATATGGCCACCAAGGCTCGGGT
**C-4365**	SNX9_SH3 in pSL4 her	GATGATGGTGGTGATGGTGCTCGAGTGCGGCCGCAAGCTTACCAGTTGCTGGTCCTTGGTA
**C-4428**	P1 Dead her	CGTGGGTCGCCGGACGCTTCGCGTTCGCGCCACCGGTGGTCGCAACCGCAACAGCCGCAACGGTCGCAACAATGGTCGCGCCGCTCGGGCTTTGCATC
**C-4429**	P2 Dead hin	CGGTGGCGCGAACGCGAAGCGTCCGGCGACCCACGGTAAGGGTGTTGCGGCGCAGGTTGCGACCGCGGGTAGCAGCAG
**C-4430**	677co in pSL4 hin	AAATAATTTTGTTTAACTTTAAGAAGGAGATATACATATGGGTATCAACCCGAGCGGCA
**C-4483**	pSL4 CRIB short For	TCCAGAAATCACAACAAACAGGTTTTATAGTTCACAAGTCGCAGATATTGGAACACCAAGTA
**C-4484**	pSL4 CRIB short Rev	GTCCAATGTGCTGGAAATTACTTGGTGTTCCAATATCTGCGACTTGTGAACTATAAAACCTG
**C-4489**	pKM431/436 sirGFP hin	CCTCTAGAAATAATTTTGTTTAACTTTAAGAAGGAGATATATGTCTGAAAAAAGACGAGATGC
**C-4490**	pKM431/436 sirGFP her	GCACCACCCCGGTGAACAGCTCCTCGCCCTTGCTCACCATTGAACTACCACTTGATCCCTCGAGTGCGGCCGCAAGCT
**C-4581**	SH3 in pDS94 hin	CTAGAAATAATTTTGTTTAACTTTAAGAAGGAGATATACCATGGCCACCAAGGCTCGGGT
**C-4582**	SH3 in pDS94 her	ACCCCGGTGAACAGCTCCTCGCCCTTGCTCACCATTGAACTACCACTTGATCCAAGCTTACCAGTTGCTGGTCC
**C-4565**	pFK31_BR-Crib siGFP in pDS94 rvs	GCACCACCCCGGTGAACAGCTCCTCGCCCTTGCTCACCATTGAACTACCACTTGATCCTGCTTGCCTTCGGAGTTC

Supplementary Table 4. Plasmids

Name	Reference
pSL4	Luczak S. Dissertation[18]
pDS94	This plasmid is based on: pET His6 GFP TEV LIC cloning vector (1GFP), gifted from Scott Gradia (Addgene plasmid # 29663; http://n2t.net/addgene:29663; RRID:Addgene_29663) and was equipped with a 10x C-terminal His-tag and a CEN/ARS/TRP cassette

Supplementary References

1 Blanchet, C. E. *et al.* Versatile sample environments and automation for biological solution X-ray scattering experiments at the P12 beamline (PETRA III, DESY). *J Appl Crystallogr* **48**, 431-443 (2015).
2 Jantos, C. A., Heck, S., Roggendorf, R., Sen-Gupta, M. & Hegemann, J. H. Antigenic and molecular analyses of different Chlamydia pneumoniae strains. *J. Clin. Microbiol.* **35**, 620-623 (1997).
3 Porod, G. Die Röntgenkleinwinkelstreuung Von Dichtgepackten Kolloiden Systemen - 1 Teil. *Kolloid-Zeitschrift and Zeitschrift Fur Polymere* **124**, 83-114 (1951).
4 Fischer, H., de Oliveira Neto, M., Napolitano, H. B., Polikarpov, I. & Craievich, A. F. Determination of the molecular weight of proteins in solution from a single small-angle X-ray scattering measurement on a relative scale. *Journal of Applied Crystallography* **43**, 101-109 (2010).
5 Rambo, R. P. & Tainer, J. A. Accurate assessment of mass, models and resolution by small-angle scattering. *Nature* **496**, 477-481 (2013).
6 Hajizadeh, N. R., Franke, D., Jeffries, C. M. & Svergun, D. I. Consensus Bayesian assessment of protein molecular mass from solution X-ray scattering data. *Scientific Reports* **8**, 7204 (2018).
7 Kikhney, A. G., Borges, C. R., Molodenskiy, D. S., Jeffries, C. M. & Svergun, D. I. SASBDB: Towards an automatically curated and validated repository for biological scattering data. *Protein Sci* **29**, 66-75 (2020).
8 Manalastas-Cantos, K. *et al.* ATSAS 3.0: expanded functionality and new tools for small-angle scattering data analysis. *J Appl Crystallogr* **54**, 343-355 (2021).
9 Panjkovich, A. & Svergun, D. I. CHROMIXS: automatic and interactive analysis of chromatography-coupled small angle X-ray scattering data. *Bioinformatics* (2017).
10 Konarev, P. V., Volkov, V. V., Sokolova, A. V., Koch, M. H. J. & Svergun, D. I. PRIMUS: a Windows PC-based system for small-angle scattering data analysis. *Journal of Applied Crystallography* **36**, 1277-1282 (2003).
11 Svergun, D. I. Determination of the Regularization Parameter in Indirect-Transform Methods Using Perceptual Criteria. *Journal of Applied Crystallography* **25**, 495-503 (1992).
12 Petoukhov, M. V. *et al.* New developments in the ATSAS program package for small-angle scattering data analysis. *J Appl Crystallogr* **45**, 342-350 (2012).
13 Bernado, P., Mylonas, E., Petoukhov, M. V., Blackledge, M. & Svergun, D. I. Structural characterization of flexible proteins using small-angle X-ray scattering. *J Am Chem Soc* **129**, 5656-5664 (2007).
14 Tria, G., Mertens, H. D., Kachala, M. & Svergun, D. I. Advanced ensemble modelling of flexible macromolecules using X-ray solution scattering. *IUCrJ* **2**, 207-217 (2015).
15 Svergun, D., Barberato, C. & Koch, M. H. J. CRYSOL– a Program to Evaluate X-ray Solution Scattering of Biological Macromolecules from Atomic Coordinates. *Journal of Applied Crystallography* **28**, 768-773 (1995).
16 Franke, D., Jeffries, C. M. & Svergun, D. I. Correlation Map, a goodness-of-fit test for one-dimensional X-ray scattering spectra. *Nat Methods* **12**, 419-422 (2015).
17 PyMOL. The PyMOL Molecular Graphics System, Version 2.0 Schrödinger, LLC. (2015).
18 Luczak, S. *Biochemical and Biophysical Characterization of the Adhesin and Invasin Pmp21 from Chlamydia pneumoniae* PhD thesis, Heinrich Heine University, Düsseldorf, (2017).

Uncropped Scans of gels and blots

Uncropped gel to Supplementary Figure 1a

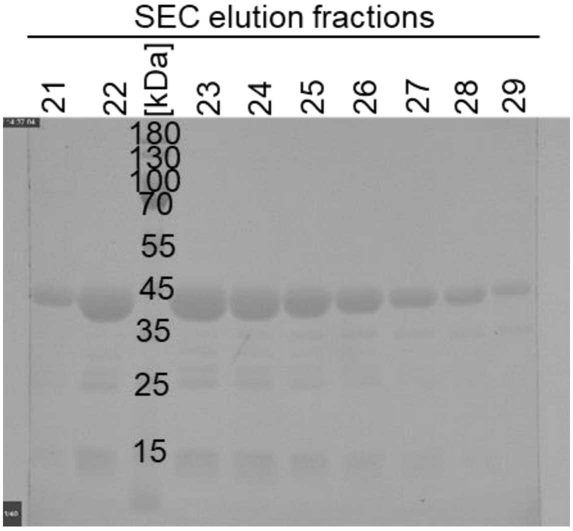

Uncropped gel to Supplementary Figure 2a

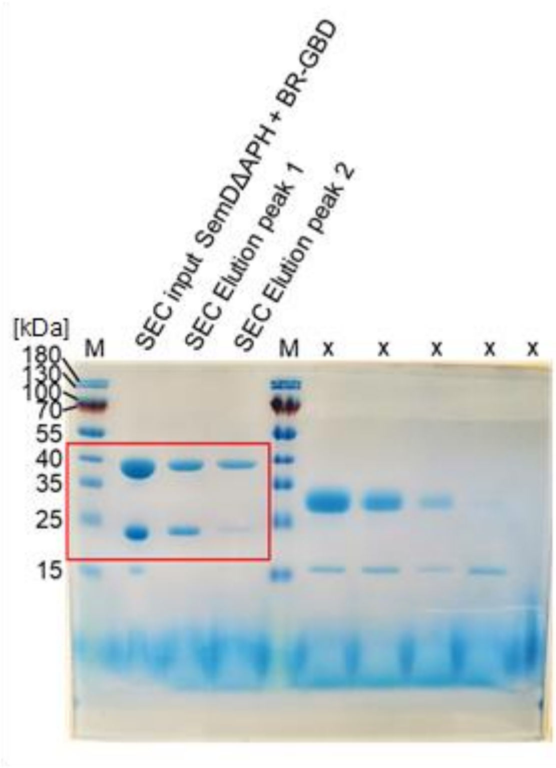

Uncropped gel to Supplementary Figure 5

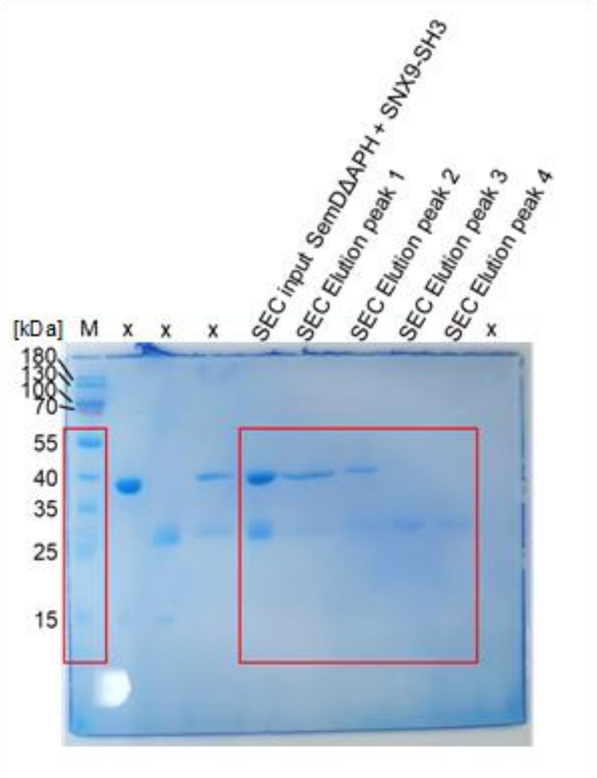

Supplement: Supplementary file 1 — Supplementary Information [file 41467_2024_51681_MOESM1_ESM.pdf]
